# Additive Effects of the Risk Alleles of *PNPLA3* and *TM6SF2* on Non-alcoholic Fatty Liver Disease (NAFLD) in a Chinese Population

**DOI:** 10.3389/fgene.2016.00140

**Published:** 2016-08-02

**Authors:** Xiaoliang Wang, Zhipeng Liu, Kai Wang, Zhaowen Wang, Xing Sun, Lin Zhong, Guilong Deng, Guohe Song, Baining Sun, Zhihai Peng, Wanqing Liu

**Affiliations:** ^1^Department of General Surgery, Shanghai General Hospital, Shanghai Jiao Tong University School of Medicine, ShanghaiChina; ^2^Department of Medicinal Chemistry and Molecular Pharmacology, College of Pharmacy, Purdue University, West Lafayette, INUSA; ^3^Department of General Surgery, Children’s Hospital of Zhengzhou, ZhengzhouChina; ^4^Department of Geriatrics, Shanghai General Hospital, Shanghai Jiao Tong University School of Medicine, ShanghaiChina

**Keywords:** additive effects, *PNPLA3*, *TM6SF2*, NAFLD, Chinese

## Abstract

Recent genome-wide association studies have identified that variants in or near *PNPLA3, NCAN, GCKR, LYPLAL1*, and *TM6SF2* are significantly associated with non-alcoholic fatty liver disease (NAFLD) in multiple ethnic groups. Studies on their impact on NAFLD in Han Chinese are still limited. In this study, we examined the relevance of these variants to NAFLD in a community-based Han Chinese population and further explored their potential joint effect on NAFLD. Six single nucleotide polymorphisms (SNPs) (*PNPLA3* rs738409, rs2294918, *NCAN* rs2228603, *GCKR* rs780094, *LYPLAL1* rs12137855, and *TM6SF2* rs58542926) previously identified in genome-wide analyses, to be associated with NAFLD were genotyped in 384 NAFLD patients and 384 age- and gender-matched healthy controls. We found two out of the six polymorphisms, *PNPLA3* rs738409 (OR = 1.52, 95%CI: 1.19–1.96; *P* = 0.00087) and *TM6SF2* rs58542926 (OR = 2.11, 95%CI: 1.34–3.39; *P* = 0.0016) are independently associated with NAFLD after adjustment for the effects of age, gender, and BMI. Our analysis further demonstrated the strong additive effects of the risk alleles of *PNPLA3* and *TM6SF2* with an overall significance between the number of risk alleles and NAFLD (OR = 1.64, 95%CI: 1.34–2.01; *P* = 1.4 × 10^-6^). The OR for NAFLD increased in an additive manner, with an average increase in OR of 1.52 *per* additional risk allele. Our results confirmed that the *PNPLA3* and *TM6SF2* variants were the most significant risk alleles for NAFLD in Chinese population. Therefore, genotyping these two genetic risk factors may help identify individuals with the highest risk of NAFLD.

## Introduction

Non-alcoholic fatty liver disease (NAFLD) is the most common liver disorder, whose prevalence is estimated to be 20–30% in Western industrialized countries (Bedogni et al., 2005). Although it is relatively less prevalent in China, the number of patients suffering NAFLD has approximately doubled in the past two decades (Fan, 2013).

The first genome-wide association study (GWAS) of NAFLD performed by Romeo et al. (2008) identified that rs738409 G allele of the *patatin-like phospholipase domain-containing-3* (*PNPLA*3) is significantly associated with increased hepatic fat levels and susceptibility to NAFLD. Subsequent analyses demonstrated that *PNPLA3* rs738409 is also associated with disease severity and progression (Sookoian and Pirola, 2011). A recent study demonstrated that the rs2294918 variant of the *PNPLA3* gene is also significantly associated with hepatic steatosis in an Italian population, independent of rs738409 (Donati et al., 2016). Besides the variants associated with steatosis, Chalasani et al. (2010) identified several genetic variants associated with histologic features of NAFLD, including NAS score, degree of fibrosis, lobular inflammation, and serum levels of alanine aminotransferase. A subsequent study in a cohort of European ancestry led by the GOLD Consortium identified four additional NAFLD risk factors located in or near neurocan gene (*NCAN*), glucokinase regulatory protein gene (*GCKR*), lysophospholipase-like 1 gene (*LYPLAL1*), and protein phosphatase 1 regulatory subunit 3b (*PPP1R3B*).(Speliotes et al., 2011). Two subsequent studies aimed to verify these findings in multiethnic cohorts. Palmer et al. (2013) tested whether these European ancestry NAFLD-related variants were associated with computed tomography defined hepatic steatosis in African- and/or Hispanic-Americans. The results indicated that allele frequency and effect size varies in different ethnic groups. Another study led by Hernaez et al. (2013) investigated the association between these variants and ultrasound-measured hepatic steatosis in non-Hispanic white (NHW), non-Hispanic black (NHB), and Mexican American (MA) participants in National Health and Nutrition Examination Survey III (NHANES III). The results also showed the variance of allele frequency and effect size across ancestries (Hernaez et al., 2013). Most recently, a new variant rs58542926 in the transmembrane 6 superfamily member 2 (*TM6SF2*) gene was associated with higher liver fat through an exome-wide association study (Kozlitina et al., 2014). A multiethnic study consisting of 957 obese children and adolescents showed that the allele frequency and effect size of *TM6SF2* rs58542926 in conferring NAFLD risk varies in Caucasians, African Americans, and Hispanics (Goffredo et al., 2016). Therefore, it is worthwhile examining the association of these variants with NAFLD in different ethnic settings. In particular, how these variants interact with each other and contribute together to the NAFLD susceptibility among each ethnic group remains to be explored.

East Asian populations account to the largest proportion of current total number of world living humans. However, they are among the least studied populations in terms of the genetic basis of NAFLD, which is incongruent with the fact that NAFLD is gaining prevalence in East Asian populations. In the present study, we aimed to evaluate the association of six previously identified polymorphisms, *PNPLA3* rs738409, rs2294918, *NCAN* rs2228603, *GCKR* rs780094, *LYPLAL1* rs12137855, and *TM6SF2* rs58542926, with NAFLD in a community-based Han Chinese population. Additionally, we analyzed the joint effects of these genetic variants on the disease risk of NAFLD.

## Materials and Methods

### Subjects

The characteristics of subjects enrolled in this study has been described before (Wang et al., 2015). Briefly, our study included 384 ultrasound (US) diagnosed NAFLD patients and 384 age- and gender-matched healthy controls from the same geographic areas as the patients. All the subjects were recruited from individuals conducting health examination in the Shanghai Jiao Tong University Affiliated First People’s Hospital (Shanghai, China). The mean age of both case and control groups was 45±13 years old, and both of them were consisted of 229 males and 155 females. Subjects with a history of heavy alcohol consumption (>20 g/day) and other specific diseases that could contribute to fatty liver, such as hepatitis B and C virus infection, drug-induced liver disease, were excluded from the study. US-based NAFLD diagnoses were made by one experienced operator without knowing clinical and biochemical evaluations of the participants. The guidelines used for diagnosis were adopted from the Chinese National Consensus Workshop on NAFLD (Zeng et al., 2008). Patients with moderate to severe fatty liver diseases were selected in order to increase the diagnostic accuracy and reliability (Hernaez et al., 2011). All subjects were unrelated and self-reported Han Chinese living in the Shanghai metropolitan area. The study conforms to the principles of the Declaration of Helsinki. All the participants have given their written informed consent, and the study method was approved by the ethical committee of the Shanghai Jiao Tong University.

### Genotyping

Genomic DNA was extracted from whole blood of each participant according to the manufacturer’s instructions (Qiagen, CA, USA). Genotyping was conducted using a Taqman-based assay (Life Technologies, CA, USA) according to the manufacturer’s instructions. Note that the *TM6SF2* rs58542926 polymorphism has been genotyped in these samples in our previous study (Wang et al., 2015). Hardy-Weinberg equilibrium (HWE) was tested for each SNP using the program PLINK (Version 1.07) (Purcell et al., 2007). The *PPP1R3B* rs4240624 was genotyped in our cohort. However, we found that the Taqman-based genotyping of this SNP does not follow the HWE given the deviated genotype distribution in our samples (*P* = *3*.592×10^-49^). We further sequenced the DNA fragment containing this SNP in 20 randomly chosen samples. All these samples demonstrated monomorphic at this variant site, suggesting that this SNP may be a fake variant. We therefore removed this SNP from our analysis. The information of allele frequencies in East Asians and other populations was obtained from The 1000 Genomes Project (Genomes Project et al., 2015).

### Statistical Analysis

Chi-square test was used for the comparisons of allele and genotype frequencies between NAFLD patients and healthy controls. Multivariable analysis was used to assess the independent effect of each SNP on the prevalence of NAFLD by incorporating all six SNPs in a multiple logistic regression model with adjustment for age, gender, and BMI, under an assumption of an additive genetic model for each SNP, in which genotypes were coded as 0, 1, or 2 according to the number of minor allele for a specific individual. Odds ratio (OR) and 95% confidence interval (CI) were calculated in groups with different number of risk alleles, and Chi-square test was used for linear trend analysis. The group with 0 risk allele was set as reference group. The proportion of narrow sense heritability explained by susceptibility variants was estimated based on multifactorial liability threshold model (So et al., 2011). The median prevalence of NAFLD in China and narrow sense heritability used for estimation is 15% (Fan, 2013) and 38.6% (Schwimmer et al., 2009), respectively. All the statistical analyses were performed using statistical package R (version 3.2.1). *P* values less than 0.05 were considered statistically significant.

## Results

### Genetic Association between SNPs and NAFLD

Genotyping was performed in 384 NAFLD patients and 384 age- and gender-matched healthy controls. The genotyping call rates for each SNP were 98.7% (*PNPLA3* rs738409), 98.9% (*PNPLA3* rs2294918), 97.9% (*NCAN* rs2228603), 99.3% (*GCKR* rs780094), 96.1% (*LYPLAL1* rs12137855), and 95.4% (*TM6SF2* rs58542926). The genotype distribution of all SNPs were in HWE in both case and control groups (all *P* > 0.05, data not shown). As shown in **Table 1**, the minor allele frequency (MAF) of SNPs genotyped in our study were comparable to the overall frequency in East Asians reported in The 1000 Genomes Project (Genomes Project et al., 2015).

**Table 1:** Minor Allele Frequency (MAF) in the present study and other populations*.

Variant	Present study	AFR	AMR	EAS	EUR
*PNPLA3* rs738409	0.38	0.12	0.48	0.35	0.23
*PNPLA3* rs2294918	0.19	0.10	0.21	0.18	0.37
*NCAN* rs2228603	0.11	0.01	0.02	0.06	0.07
*GCKR* rs780094	0.45	0.13	0.36	0.48	0.41
*LYPLAL1* rs12137855	0.055	0.18	0.15	0.08	0.21
*TM6SF2* rs58542926	0.067	0.02	0.06	0.09	0.07

*Allele frequencies information was collected from 1000 Genomes Project (Genomes Project et al., 2015). AFR, African; AMR, American; EAS, East Asian; EUR, European.

The genotype distributions of these SNPs and their association with NAFLD are listed in **Table 2**. Note that the association between *TM6SF2* rs58542926 polymorphism and NAFLD in these samples have been reported in our previous study (Wang et al., 2015). Two out of six variants, *PNPLA3* rs738409 and *TM6SF2* rs58542926, were significantly associated with NAFLD. The frequency of G allele of the *PNPLA3* rs738409 variant was significantly higher in NAFLD patients compared to those in healthy controls (OR = 1.48, 95%CI: 1.20–1.82; *P* = 0.00023), and *PNPLA3* GC and GG genotypes were significantly associated with increased risk of disease (GC: OR: 1.52, 95%CI: 1.11–2.07; *P* = 0.008; GG: OR: 2.24, 95%CI: 1.41–3.55; *P* = 0.00055). Similarly, T allele of *TM6SF2* rs58542926 was more prevalent in the subjects with NAFLD (OR: 2.06, 95%CI: 1.34–3.17; *P* = 0.00086). The rest of SNPs, *PNPLA3* rs2294918, *GCKR* rs780094, *NCAN* rs2228603, and *LYPLAL1* rs12137855 did not show any independent significant association with NAFLD in the population.

**Table 2:** Genotype distribution and MAF of *PNPLA3* rs738409, rs2294918, *NCAN* rs2228603, *GCKR* rs780094, *LYPLAL1* rs12137855, and *TM6SF2* rs58542926.

Genotype	NAFLD	Healthy	*P*-value*	OR (95% CI)
***PNPLA3* rs738409**				
CC	122	169		Ref
GC	191	174	0.0080	1.52 (1.11, 2.07)
GG	63	39	0.00055	2.24 (1.41, 3.55)
MAF (G allele)	0.42	0.33	0.00023	1.48 (1.20, 1.82)
***PNPLA3* rs2294918**				
GG	258	240		Ref
GA	114	124	0.32	0.86 (0.63, 1.17)
AA	9	15	0.17	0.56 (0.24, 1.30)
MAF (A allele)	0.17	0.20	0.14	1.22 (0.94, 1.57)
***NCAN* rs2228603**				
CC	299	304		Ref
CT	70	70	0.92	1.02 (0.70, 1.47)
TT	9	0	–	–
MAF (T allele)	0.12	0.093	0.15	0.78 (0.56, 1.09)
***GCKR* rs780094**				
CC	123	110		Ref
CT	189	182	0.65	0.93 (0.67, 1.29)
TT	68	91	0.051	0.67 (0.45, 1.00)
MAF (T allele)	0.43	0.48	0.062	1.21 (0.99, 1.48)
***LYPLAL1* rs12137855**				
CC	333	325		Ref
TC	44	35	0.39	1.23 (0.77, 1.96)
TT	0	1	–	–
MAF (T allele)	0.058	0.051	0.55	0.87 (0.56, 1.37)
***TM6SF2* rs58542926**				
CC	302	333		Ref
CT	65	33	0.00054	2.17 (1.39, 3.40)
TT	0	0	–	–
MAF (T allele)	0.089	0.045	0.00086	2.06 (1.34, 3.17)

*Differences between genotype frequencies were tested using Chi-square test. *P* < 0.05 was considered to be statistically significant. MAF, minor allele frequency; NAFLD, non-alcoholic fatty liver disease; OR, odds ratio; CI, confidence interval.

### Independent Effect of SNPs on NAFLD

In order to investigate the independent effect of each variant on the susceptibility to NAFLD, all the six SNPs were included in the multiple logistic regression model with age, gender, and BMI as covariates. As shown in **Table 3**, *PNPLA3* rs738409 and *TM6SF2* rs58542926 variants were significantly associated with NAFLD after adjustment for age, gender and BMI (*P* = 0.00087 and 0.0016, respectively).

**Table 3:** Association between *PNPLA3* rs738409, rs2294918, *NCAN* rs2228603, *GCKR* rs780094, *LYPLAL1* rs12137855, and *TM6SF2* rs58542926 variants and NAFLD*.

Variant	Coefficient estimate	Standard error	OR (95%CI)	*P*-value
*PNPLA3* rs738409	0.42	0.13	1.52 (1.19,1.96)	0.00087
*PNPLA3* rs2294918	0.024	0.15	1.02 (0.76,1.39)	0.88
*NCAN* rs2228603	0.13	0.19	1.14 (0.79,1.65)	0.49
*GCKR* rs780094	0.20	0.11	1.23 (0.99,1.53)	0.068
*LYPLAL1* rs12137855	-0.038	0.25	0.96 (0.59,1.57)	0.88
*TM6SF2* rs58542926	0.75	0.24	2.11 (1.34,3.39)	0.0016

*Associations were tested using logistic regression with adjustment for age, gender, and BMI. OR, odds ratio; CI, confidence interval.

### Additive Effects of the Risk Alleles of *PNPLA3* rs738409 and *TM6SF2* rs58542926 in NAFLD

**FIGURE 1:**
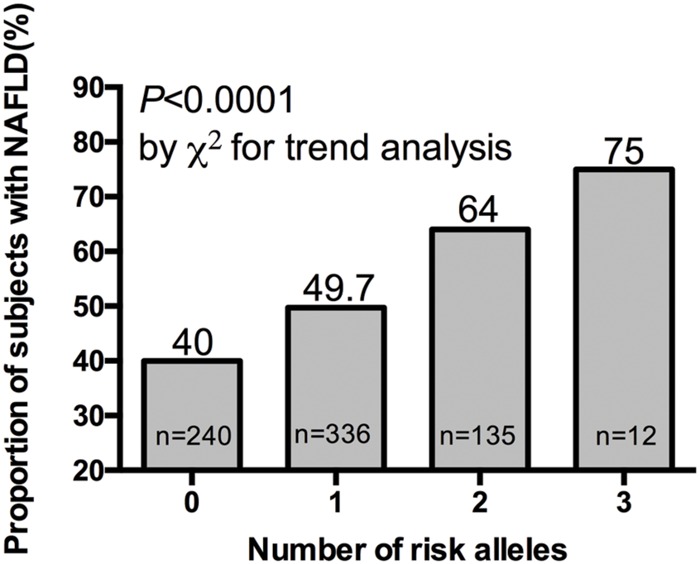
**Proportion of NAFLD patient in 40, 336, 135, and 12 subjects harboring 0, 1, 2, and 3 risk alleles respectively**.

**FIGURE 2:**
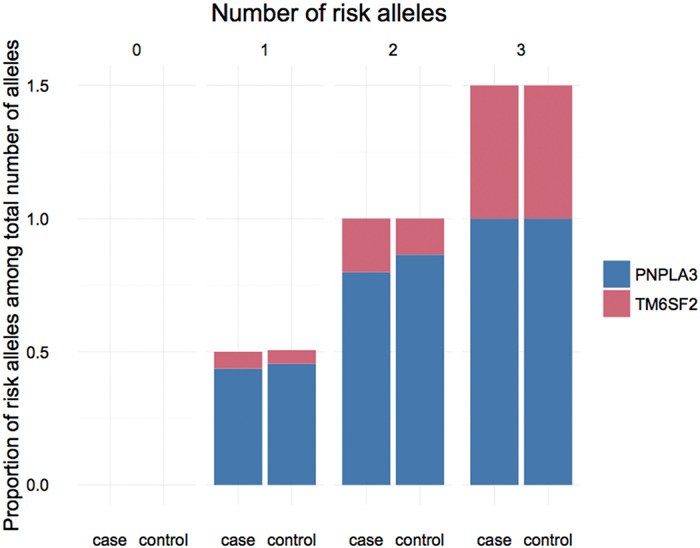
**Proportion of PNPLA3 and TM6SF2 risk allele in NAFLD patients and healthy controls carrying different number of risk alleles**.

**FIGURE 3:**
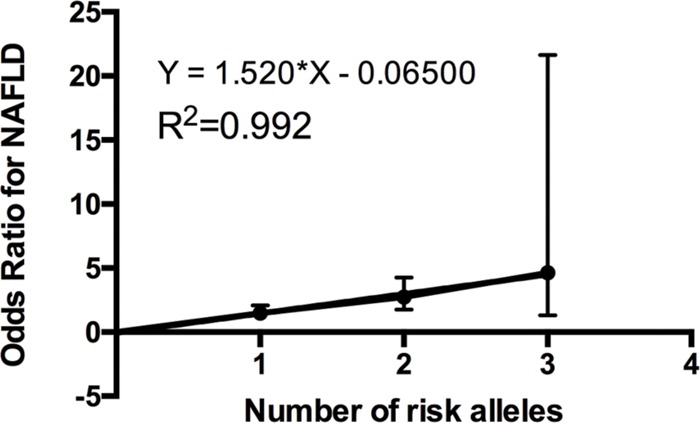
**Odds ratio (ORs) (with 95% CI) for developing NAFLD is a linear function of the number of risk alleles.** ORs obtained by multiple logistic regression after adjustment for age, gender and BMI. The actual odds ratio numbers are as follows: 0 (reference group OR = 1), 1 [OR = 1.48 (1.06–2.08)], 2 [OR = 2.73 (1.77–4.26)], and 3 [OR = 4.65 (1.32–21.65)].

We further investigated the joint effect of the risk allele of *PNPLA3* rs738409 and *TM6SF2* rs58542926 on the risk of NAFLD. **Figure 1** shows the proportion of NAFLD patients in the individuals harboring different number of risk alleles. Ninety-six NAFLD patients did not harbor any risk alleles, and we did not observe any individuals with four risk alleles in our study. The proportion of NAFLD patients increased significantly (*P* < 0.0001, Chi-square test for trend) along with the increase of the number of risk alleles. The overall significance between the number of risk alleles and NAFLD incidence is high, with a *P* value of 1.4×10^-6^, using a logistic regression model after adjustment for age, gender and BMI. We also plotted the proportion of each risk allele for healthy controls and NAFLD patients separately (**Figure 2**). **Figure 3** shows the OR for NAFLD in the group of patients with different number of risk alleles, using the individuals with zero risk allele as the reference group. As shown in the **Figure 3**, the risk of NAFLD increases linearly with the number of risk alleles with an average of 1.52-fold increase in OR for each additional risk allele. The regression line fit excellently for the data with the R squared value of 0.992.

## Discussion

Using a cohort of 384 NAFLD patients and 384 age- and gender-matched healthy controls, we confirmed that *PNPLA3* rs738409 and *TM6SF2* rs58542926 were independent risk factors conferred susceptibility to NAFLD in a Han Chinese population. Moreover, we examined the joint effects of *PNPLA3* rs738409 and *TM6SF2* rs58542926 polymorphisms. To our best of knowledge, this is the first study to report the additive effect of *PNPLA3* rs738409 and *TM6SF2* rs58542926 polymorphisms on NAFLD in a Chinese population.

*PNPLA3* rs738409 was the first identified genetic variant that contributes to the differences in liver fat levels and the susceptibility to NAFLD (Romeo et al., 2008). Results in our study confirmed the association between *PNPLA3* rs738409 and increased risk for NAFLD in a Han Chinese population, which emphasized its effects on NAFLD across multiple ethnic groups. *TM6SF2* rs58542926 was the most recently identified genetic variant to be associated with NAFLD (Kozlitina et al., 2014). The subsequent studies demonstrated that this variant was also associated with the progression of NAFLD disease (Liu et al., 2014; Dongiovanni et al., 2015). *TM6SF2* rs58542926 was initially identified in a multiethnic cohort consisting of African Americans, European Americans, and Hispanics. In our previous study, we first reported *TM6SF2* rs58542926 polymorphism as an independent risk factor that contributes to NAFLD in a Chinese population (Wang et al., 2015). In the current study, we further investigated its joint effects with *PNPLA3* rs738409 on the risk of developing NAFLD. Notably, the association between the number of risk alleles and NAFLD incidence is very significant, with a beta value of 1.52 and the mean OR of 4.65 in the individuals carrying three risk alleles, suggesting the value of early diagnosis of NAFLD by genotyping these two variants. An additive model of risk alleles was shown to fit very well in explaining the greater probability of NAFLD. Our findings in the Chinese population are consistent with previous study performed in other ethnic groups. Goffredo et al. (2016). have shown that the hepatic fat fraction increased as the sum of risk allele in *TM6SF2* rs58542926, *PNPLA3* rs738409, and *GCKR* rs1260326 in an obese children cohort including Caucasians, African Americans, and Hispanics. Our study confirmed this joint effect in a Chinese population, suggesting a new perspective in NAFLD classification and diagnosis using risk allele number counting. Moreover, the genetic variability in *PNPLA3* rs738409, in *TM6SF2* rs58542926, and in the combined effects explained 8.2, 9.6, and 19.2% of the heritability of NAFLD in Chinese population, respectively. This indicates additional possible unknown risk variants yet to be identified among Chinese NAFLD patients.

PNPLA3, which encodes a 481-residue protein, exhibits lipase activity against triglyceride (TG) in hepatocytes and retinyl esters in hepatic stellate cells (He et al., 2010; Huang et al., 2011; Pirazzi et al., 2014). Missense mutation (I148M) in PNPLA3 leads to a loss of function and thus promotes hepatic steatosis by limiting triglyceride hydrolysis (He et al., 2010; Smagris et al., 2015). Although the specific function of TM6SF2 is still unknown, Kozlitina et al. (2014) found that the Glu167Lys variant led to a lower levels of protein expression than wild-type TM6SF2 in cultured hepatocytes. A subsequent functional study indicated that the inhibition of TM6SF2 protein expression results in reduced secretion of TG-rich lipoproteins (TRLs), which increases cellular TG concentration (Mahdessian et al., 2014). Therefore, these two variants seem to affect the homeostasis processes of hepatic fat separately, whose functions are relatively independent but synergistically involved in the development of hepatic steatosis. Our results of the additive effects of the risk alleles of PNPLA3 and TM6SF2 on NAFLD were consistent with their biological functions, which highlights the possibility to identify high risk individuals by genotyping these two risk factors.

Inconsistent with the previous finding that *GCKR* rs780094 significantly increased the risk of NAFLD in obese Chinese children (Lin et al., 2014), we did not observe significant association between this variant and NAFLD. Given that the sample size and alleles frequencies of our study (*n* = 766, MAF = 0.45) were comparable to the previous study (*n* = 797, MAF = 0.49) (Lin et al., 2014), we speculated that *GCKR* rs780094 may only have a moderate effect on NAFLD incidence in Chinese population. On the other hand, participants enrolled in our study were adults with a mean age of 45 years old, which introduces more possible influence of environment in the study. The lack of consistency for *GCKR* variant suggests its potential interaction with environment. Similar to the results reported in the previous study (Lin et al., 2014), we did not find significant associations between either *NCAN* rs2228603 or *LYPLAL1* rs12137855 and NAFLD in Han Chinese population. Recent studies have shown that conditioning on the *TM6SF2* variant abolishes the association between *NCAN* variant and NAFLD while the reverse does not occur (Kozlitina et al., 2014), suggesting *NCAN* variant is not the causal variant associated with NAFLD. Our results of multivariable analysis confirmed this finding by exploring the independent effects of individual variant on NAFLD after conditioning on all the other SNPs. With regard to *LYPLAL1* rs12137855 variant, as shown in the **Table 1**, the MAF of *LYPLAL1* rs12137855 in East Asians is much lower than those in other populations, which reduces the power to detect the association given that our sample size is relatively small. Of course, this could be also due to the limited effect size of this variant in a Chinese population.

The NAFLD phenotype in our study was defined using an US-based diagnosis, which might cause concern about whether it is appropriate to detect NAFLD. However, according to a meta-analysis comparing the diagnostic accuracy and reliability of the US-based method to histology-based method (gold standard), the US-based method was fairly good in detecting moderate-severe fatty liver (Hernaez et al., 2011), and multiple genetic studies using the US-based diagnosis method have successfully demonstrated the associations between genetic polymorphisms and NAFLD (Hernaez et al., 2013; Sookoian et al., 2015).

## Conclusion

We confirmed the associations between genetic variants in *PNPLA3* and *TM6SF2* genes and NAFLD risk in a Han Chinese population. We also demonstrated the additive effects of these two genetic risk factors on conferring risk of NAFLD in Han Chinese. The differences in allele frequencies of several other risk alleles that were identified in other populations but are not associated with NAFLD in Chinese indicates that additional unknown risk alleles are yet to be identified in Han Chinese populations.

## Author Contributions

XW sample collection, genotyping, data collection, manuscript drafting; ZL data analysis and manuscript drafting; KW sample collection, genotyping, and data collection; ZW, XS, LZ, GD, GS, and BS participated sample and data collection; ZP: coordinated and directed the study; WL conceived the study, data analysis, manuscript drafting.

## Conflict of Interest Statement

The authors declare that the research was conducted in the absence of any commercial or financial relationships that could be construed as a potential conflict of interest. The reviewer BL and handling Editor declared their shared affiliation, and the handling Editor states that the process nevertheless met the standards of a fair and objective review.
